# Reviewers Acknowledgement

**DOI:** 10.1002/1878-0261.13809

**Published:** 2025-02-04

The Editors of *Molecular Oncology* would like to thank all referees who have given their time and expertise to review articles submitted for publication in Volume 18. *Molecular Oncology* heavily relies on reviewers' careful assessment, constructive criticism, and timely response to ensure both quality and fast turnaround times.

The names of these reviewers are listed below; we apologize if we have inadvertently omitted anyone.

Abdul Rahman Amirah

Aceto Nicola

Afflerbach Ann‐Kristin

Ahmed A.U.

Ailles Laurie

Aird Katherine

Akbari Mohsen

Ali Ahmed

Almeida Raquel

Al‐Shaheri Fawaz N.

Altomare Deborah A.

Altrock Eva

Alvarez‐Fernandez Monica

Amantini Consuelo

Ambrogio Chiara

Andersen Bogi

Andersen Claus

Angeli Jose Pedro Friedmann

Anh Nguyen Hoang

Annibali Daniela

Antona Annamaria

Arica Betul

Ariey‐Bonnet Jérémy

Arik Sever Elif

Arreguin Karina

Awah Chidiebere

Awasthi Deepika

Azmi Asfar

Baba Hideo

Babačić Haris

Bagchi Anindya

Baker Kristi

Baldassarre Gustavo

Baltanas Fernando C.

Bansal Ruchi

Barbagallo Davide

Barderas Rodrigo

Bartek Jiri

Bartsch Jörg Walter

Barzegar Behrooz Amir

Basrai Munira

Bedogni Barbara

Beggs Andrew

Bemanian Vahid

Ben‐David Yaacov

Benitez Riquelme Diego

Bernards Rene

Bernassola Francesca

Berns Kathrin

Bharadwaj Alamelu G.

Bhattacharya Rajat

Bijlsma Maarten

Birkbak Nicolai Juul

Bitler Benjamin G.

Blandino Giovanni

Boccaccio Carla

Bochtler Tilmann

Bockmayr Michael

Bogeski Ivan

Bohaciakova Dasa

Bonfiglio Rita

Bonnet Dominique

Bonora Elena

Bose Mukulika

Boucai Laura

Boulagnon‐Rombi Camille

Boye Kjetil

Bragoszewski Piotr

Braicu Cornelia

Brandt Burkhard

Brazil Derek Patrick

Brennen W. Nathaniel

Brix Nikko

Brizzi Maria Felice

Brummer Tilman

Brunner Thomas

Buck Stefan

Bulstrode Harry

Burma Sandeep

Bussolino Federico

Butler Lisa M.

Cai Yanyan

Caini Saverio

Campaner Stefano

Candi Eleonora

Caraglia Michele

Caratti Bozhena

Carrer Alessandro

Carrier Alice

Casiano Carlos

Castegna Alessandra

Catanzaro Giuseppina

Cavallaro Ugo

Cerezo Michaël

Chacko Jenu V.

Chaluvally‐Raghavan Pradeep

Chang Yu‐Chan

Chaveroux Cedric

Chen Chun‐Liang

Chen Danyu

Chen Weikang

Chen Xi

Chen Yinhao

Chen Yinnan

Chen Yuan‐Hao

Cheng Lixin

Cheung Lydia W.T.

Chevet Eric

Chiu H.A.

Chiu Sung

Choudhary Bibha

Christmann Markus

Cinar Bekir

Cives Mauro

Classon Johanna

Clemenceau Alisson

Cloos Jacqueline

Clynes Martin

Cooke Mariana

Cordelier Pierre

Cornish Toby

Coronas Valerie

Cottard Félicie

Cottu Paul H.

Courjaret Raphael

Coutts Amanda

Crimini Edoardo

Cristofanilli Massimo

Cristofari Gael

Cross Ryan S.

Cucu Dana

Cusan Monica

Da Cruz Paula Arnaud

Dahlhoff Maik

Damia Giovanna

Dasgupta Nirmalya

Davidson Irwin

Davis Anthony

Dazert Eva

De La Cruz Vargas Jhony Alberto

de Miguel Perez Diego

de Souza Ferreira Luiz Philipe

Deborde Sylvie

Defilippi Paola

Delgado Bellido Daniel

Derakhshan Fatemeh

Dhiman Alisha

Di Nicolantonio Federica

Dickerson Erin

Didonato Marzia

Dimas Konstantinos

DiPersio John

Dirix Luc Y.

Dive Caroline

Doberstein Kai

Doello Kevin

Dolci Susanna

Donnadieu Emmanuel

Dopeso Higinio

Doronzo Gabriella

Doultsinos Dimitrios

Dovedi Simon J.

Downs Jessica A.

Dragomir Mihnea Paul

Drapela Stanislav

Drapkin Ronny I.

Drosos Yiannis

Drosten Matthias

Dubois Ludwig

Dunbar Andrew

Duncan James S.

Ebeid Kareem

Ebert Gregor

Echarri Asier

Edsjo Anders

Ehata Shogo

Eickhoff Nils

Eilers Martin

Eisenman Robert

Endo M.

Erlacher Miriam

Erson‐Bensan Ayse Elif

Estephan Hala

Fabregat Isabel

Farace Francoise

Felley‐Bosco Emanuela

Feng Jifeng

Ferro Matteo

Fijneman Remond

Fischer Martin

Fisher Paul B.

Floris Giuseppe

Forster Michael

Fortunato Orazio

Foster Leonard

Fraser Michael

Fric Jan

Fritz Connor

Furney Simon J.

Furtado O'Mahony Luke

Furukawa Toru

Gabrilovich Dmitry I.

Gago Nuria

Galante Pedro A.F.

Garg Manoj

Garzoni Luciana Ribeiro

Gattei Valter

Gautier Mathieu

Gavard Julie

Gazzaniga Paola

Ghorbian Saeid

Ghosh Alok

Gibbs Peter

Gil Valdes Jeovanis

Giovannetti Elisa

Gires Olivier

Gjertsen Bjørn T.

Gkretsi Vasiliki

Goberdhan Deborah Ci

Godbout Roseline

Goldstein Alisa M.

Gorospe Myriam

Gregoricchio Sebastian

Griñan Lisón Carmen

Grossman Steven R.

Grote Alexander

Grünewald Thomas

Gu Wenjin

Guan Chen

Guerra Borja

Guerra M. Carmen

Guerrero‐Zotano Angel

Gupta Avantika

Ha Chung Shing Rex

Haendler Bernard

Hagenfeld Daniel

Haikala Heidi M.

Hallberg Bengt

Hanash S.M.

Hansen Carsten

Harada Hiroshi

Hatley Mark

Hayden Michael R.

Hazra Rasmani

Hegi Monika

Hempel Anne

Hernández‐Rivas Jesús María

Hernando Barbara

Hoare Matthew

Höglinger Carmen

Holbert Cassandra

Hoon Dave

Horak Peter

Hou Jian

Huang Suyun

Huertas Pablo

Hühn Daniela

Humphrey Tim

Hurwitz Stephanie N.

Hydbring Per

Ibáñez‐Costa Alejandro

Ibarra Luis Exequiel

Imyanitov Evgeny

Inaba Hiroaki

Innes James

Ionescu Filip

Iovanna Juan L.

Jackson Mark

Jagadeeshan Sankar

Janssen Emiel

Janssens Katleen

Ji Fen Fen

Jia Guangshuai

Jia Lintao

Jin Nenghao

Kallergi Galatea

Kanayama Mayuko

Karras Panagiotis

Karumbaiah Lohitash

Kasimir‐Bauer Sabine

Katayama Ryohei

Keane Lily

Keeshan Karen

Keller Laura

Ketteler Robin

Kidd Mark

Kietzmann Thomas

Kim Kiwook

King Michael

Kirkland Jacob

Kiss‐Toth Endre

Kitagawa Hiroshi

Kluiver Joost

Koch Claudia

Kodous Ahmad

Koksoy Ayse A.

Kolesnikova Varvara

Komatsu Masaaki

Kondo Nobuyuki

Kondo Takeshi

Kortum Robert L.

Koskinen Paivi J.

Kovacevic Zaklina

Kraan Jaco

Krause Daniela

Krenz Bastian

Kreuzaler Peter

Kruyt Frank A.E.

Kumar Sushil

Kumari Sonam

Künstner Axel

Kurelac Ivana

Kyprianou Natasha

Kyte Jon Amund

Labidi‐Galy Intidhar

Lack Nathan A.

Ladbury John E.

Ladomery Michael R.

Lamarche Nathalie

Lathia Justin Durla

Lathlean Timothy

Lau Harry

Lawlor Beth

Le Dévédec Sylvia Emmanuelle

Le Tourneau Christophe

Leszczynska Katarzyna B.

Lewis Robert

Li Bilan

Li Eric

Li Jing

Li Junling

Li Kunlun

Li Loretta S

Li Qing

Li Wenzhe

Li Yanmei

Lianidou Evi

Lin Cheng‐Wei

Lindquist Jonathan A.

Ling Yan

Liou Angela

Liu Changzheng

Liu Chuanfa

Liu Hongbo

Liu Hsiao‐Sheng

Liu Wei‐Xin

Liu Yawei

Liu Zhonghua

Lo Yuk Ming Dennis

Loeb Keith

Lohr Matthias

Lokeshwar Bal L.

Long Jaclyn

Long Nguyen Phuoc

Lonsdale Andrew

Lopez‐Contreras Andres Joaquin

Lopez‐Guerrero Jose Antonio

Lord Christopher

Lu Xiaofan

Lu Yinghong

Lu Yong‐Jie

Lum Julian J.

Luna Velez Maria Victoria

Ma Chao

Ma Haowei

Maeda Shina

Magbanua Mark

Maker Ajay V.

Makhov Peter

Makvandi Manoochehr

Malapelle Umberto

Marasca Federica

Marchini Sergio

Markkanen Enni

Martínez‐López Angélica

Martins Eduarda P.

Marzo Isabel

Masson Murielle

Maurer Tobias

McBride William

McEwan David

Medina Heidy N.

Medova Michaela

Menga Alessio

Mezzanzanica Delia

Michelena Jone

Miele Evelina

Mieruszynski Stephen

Mikheev Andrei M.

Mills Alea

Min Li

Mineo Marco

Mitani Takakazu

Molina‐Vila Miguel A.

Mondal Jayanta

Mondino Anna

Moolenaar Wouter H.

Moon Ejung

Morandi Andrea

Morello Silvana

Moreno Carlos

Morscher Raphael J.

Moss Esther L.

Muehlebner Angelika

Muiños Ferran

Muller Patricia A.J.

Munoz‐Espin Daniel

Muranen Taru

Muratani Masafumi

Müther Michael

Nadal Ernest

Nag Chaudhuri Ronita

Narita Masashi

Nasr Rihab

Nassa Giovanni

Nasser Mohd Wasim

Nathan James Alexander

Nelson Colleen

Neubauer Hans

Nigita Giovanni

Niklison‐Chirou Maria Victoria

Nishida Toshirou

Normanno Nicola

Nowak Daniel

Oh Kyu‐Young

Olcina Monica

Oliveira‐Ferrer Leticia

Oltean Sebastian

Ombrato Luigi

Origanti Sofia

Orimo Akira

Oscorbin Igor P.

Oskarsson Thordur

Österroos Albin

Pal Arnab

Palacios Jose

Pancaldi Vera

Papaccio Federica

Parisi Daniele

Paronetto Maria Paola

Paschen Annette

Peeters Rens

Pegg Anthony

Peng Yong

Pentimalli Francesca

Pepe Francesco

Peracaula Rosa

Peraldo‐Neia Caterina

Pérez de Castro Ignacio

Perkins Neil D.

Peters Fleur

Pfeffer Ulrich

Phanstiel Otto

Pierga Jean‐Yves

Pineau Pascal

Pisapia Pasquale

Politz Oliver

Pozniak Joanna

Prekovic Stefan

Pucci Perla

Radosa Julia

Rafei Hind

Rai Vikas

Rainero Elena

Ramos Kenneth S.

Rankin Erinn

Real Francisco X.

Reddy Aramati Bindu Madhava

Redmer Torben

Rehman Fawad Ur

Reitsam Nic Gabriel

Ricardo Sara

Ricciardelli Carmela

Richardson Des R.

Riediger Anja Lisa

Roberti Annalisa

Rodrigues Manuel

Rodrigues Pedro

Rodriguez‐Aguayo Cristian

Rodriguez‐Berriguete Gonzalo

Rogalla Stephan

Romero Atocha

Rosell Rafael

Rota Rosella

Rotblat Barak

Rouleau Etienne

Rowe Helen

Ruan Shaobo

Rubin Mark

Rubio‐Alarcón Carmen

Rutkowska Aleksandra

Rutkowski Piotr

Sabarinathan Radhakrishnan

Saha Biswarup

Said Neveen

Sala Gianluca

Salvatore Domenico

Sampson Nathialie

Sánchez‐Margalet Victor

Sandler Anthony David

Santamaria David

Sartori Alessandro A.

Sasaki Atsuo T.

Saur Dieter

Scaffidi Paola

Scala Stefania

Scardaci Rossella

Schindl Rainer

Schmitt Nicole C.

Schneider Pauline

Schreml Stephan

Schwaller Juerg

Secrier Maria

Segers Rony

Seki Ekihiro

Seki Naohiko

Selth Luke A.

Sena Laura

Seo Sang Uk

Seoane Jose Antonio

Serrels A.

Shachar Idit

Sher Yuh‐Pyng

Shim Gayong

Short Rob

Silwal‐Pandit Laxmi

Sirozh Oleksandra

Skotheim Rolf I.

Smirnov Artem

Smits Kim

Smits Ron M.J.M.

Smits Veronique A.J.

Snijder Berend

Sollazzo Manuela

Song Minsun

Soucek Laura

Soucek Pavel

Spick Matt

Srivastava Shiv

Stamatakis Konstantinos

Stankevicius Vaidotas

Starkova Julia

Stassi Giorgio

Steinbach Joachim

Steingrimsson Eirikur

Stoddart Angela

Stoecklein Nikolas H.

Stolfi Carmine

Storkus Walter

Strandgaard Trine

Strauss Bryan E.

Su Allen

Sumbayev Vadim

Sun Wei‐Hsin

Sun Xueqin Sherine

Sun Yi

Sundfeldt Karin

Sundvall Maria

Suzuki Rei

Szeliga Monika

Tadié Jean‐Marc

Takayama Kenichi

Takeshita Toru

Tanaka Hiroyoshi Y.

Tasken Kristin

Tattersall Luke

Taulli Riccardo

Taylor Samuel

Tee Andrew

Telange Rahul

Telleria Carlos M.

Teng Hao‐Wei

Terada Naoki

Termini Christina

Terng Harn‐Jing

Thannickal Victor J.

Thiery Jean‐Paul

Thijssen Rachel

Thomas Daniel

Thompson Alex

Timpson Paul

Toledo Rodrigo A.

Tomé Mercedes

Toretsky Jeffrey A.

Torres‐Ayuso Pedro

Tortorella Domenico

Tost Jorg

Tran Hoanh

Treps Lucas

Tress Michael

Tullo Apollonia

Urbini Milena

Urup Thomas

Vallejo Juan A.

Van Doorn Remco

Van Keymeulen Alexandra

Vanderhyden Barbara

Vazquez Miguel

Vecera Marek

Velasco Guillermo

Verdijk Robert M.

Veth Tim

Vetvik Katja

Vick Binje

Vigna Elisa

Viktorsson Kristina

Villegas Jaime

Visal Tanvi

Viswanadhapalli Suryavathi

Viswanathan Srinivas R.

von Bubnoff Nikolas

Vorobyeva Anzhelika

Wagner Michael

Wandall Hans H.

Wang Jichuang

Wang Lu

Wang Tza‐Huei

Wang Yongsheng

Weigelt Britta

Werner Stefan

Westermarck Jukka

Wiemann Stefan

Wilting Saskia M.

Wing Peter

Winter Christof

Wong Dennis

Wong Ka‐Chun

Wu Dongdong

Wu Jiayu

Wu Wei

Wyatt Alexander

Xi Qiaoran

Xiao Zhi‐Qiang

Xie Max

Xue Jing

Yamamoto Yusuke

Yang Jine

Yang Jing

Yang Zuli

Ye Jiangbin

Ylstra Bauke

Yu Xiaobo

Yu Yongfu

Zannini Mariastella

Zhang Henley

Zhang Jun

Zhao Yu‐Yue

Zheng Qinheng

Zhou Chumin

Zhou Qing

Zhou Xin

Zhou Zhihang

Zhou Qiming

Zhu Shouhai

Zienolddiny Shanbeh

Zitzelsberger Horst

Zou Yutian

